# Bilateral Cerebrovascular Stroke as an Initial Presenting Symptom of Moyamoya Disease

**DOI:** 10.1155/2018/2591494

**Published:** 2018-11-13

**Authors:** Ester Ilyayeva, Khaled Nada, Roxane Farahi Far, Kamal Albright, Manmeet Kaur Gujral, Menachem Gold

**Affiliations:** Lincoln Medical and Mental Health Center, 234 E 149Th St., Bronx, NY 10451, USA

## Abstract

Moyamoya disease is a rare condition affecting the circle of Willis and its branching arteries. While the pathogenesis is unclear, it causes progressive occlusion of multiple cerebral vessels leading to severe strokes. We report a case of a 47-year-old Hispanic woman with HTN presented with altered mental status and bilateral upper and lower extremity weakness with dystonic-like upper extremity movement. Serial brain CTs and angiography were performed which showed massive frontal and parietal cerebral infarcts with radiological evidence of moyamoya disease.

## 1. Introduction

Bilateral cerebroocclusive events occurring simultaneously are extremely rare and are often due to an alternative underlying etiology. Moyamoya is a rare progressive cerebrovascular disease that presents as a hemorrhagic or ischemic event in adults. Moyamoya is a chronic cerebral vasculopathy first described in 1957 by Takeuchi and Shimizu. It is characterized by chronic worsening occlusion of the arteries of the circle of Willis leading to the development of abnormal collateral vessels. The term “moyamoya” refers to the appearance of the collateral vessels that resemble a “puff of smoke” in Japanese. While computed tomography (CT), MRI-angiography, or ultrasonography may be used as diagnostic tools, the gold standard for visualizing the collateral circulation network characteristic of moyamoya disease is conventional angiography. Here we present a patient with bilateral infarction of the ACA and left MCA with a network of collateral vessels consistent with moyamoya disease.

## 2. Case Presentation

A 47-year-old female with a past medical history of hypertension presented to the emergency department at Lincoln Hospital, Bronx, New York, with altered mental status. As per the patient's daughter, she was found confused in the bathroom, with last well known time eight hours prior to her presentation. Upon examination in the emergency department, she appeared altered, nonverbal, but responsive to painful stimuli, involuntarily opening and closing her eyes, and able to protect her airway. The patient was afebrile, tachycardic to 130 with a blood pressure of 140/90. Neurological exam was remarkable for left gaze preference, spasticity of all extremities, bilateral lower extremity hyperreflexia, occasional myoclonus, and positive Babinski sign bilaterally. She was also noted to be moving all extremities purposelessly. All labs were normal including complete blood count, basic metabolic panel, creatinine kinase, and troponin. Serum levels of salicylate, acetaminophen, carbamazepine, lithium, valproic acid, and alcohol were negative, and urine toxicology screen was also negative for any illicit drugs. EKG showed normal sinus rhythm.

**Figure 1:**
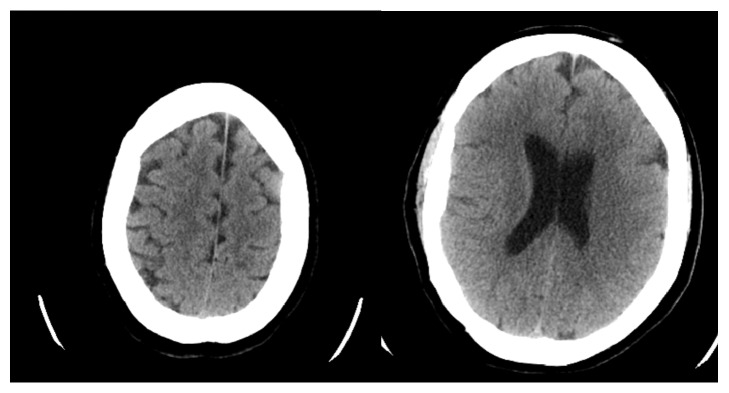
Brain CT on presentation.

**Figure 2:**
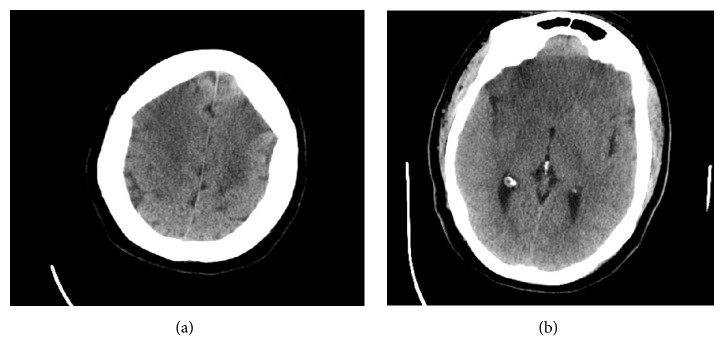
Axial CT images demonstrating bilateral large areas of hypoattenuation consistent with acute MCA and ACA infarcts.

Initial brain CT was negative for hemorrhage or any infarcts [Figure 1]. A few hours later, the patient became more lethargic, unable to protect her airway, and, given the high risk for aspiration, she was intubated and admitted to the medical ICU. Lumbar puncture was done and CSF analysis was negative for bacterial meningitis, as well as CMV, HSV PCR, toxoplasma, west Nile virus, and oligoclonal bands. She had a repeat CT the next day that demonstrated large areas of low attenuation in both cerebral hemispheres consistent with acute ACA and MCA infarcts [Figures 2(a) and 2(b)]. In view of the patient's presentation and absence of classical stroke risk factors, CT angiography and vasculitis work-up were sent including ANA, c-ANCA, p-ANCA, ESR, CRP, antiphospholipid antibodies, Factor V Leiden, prothrombin gene mutation, protein C, protein S, cryoglobulins, and complement C3 and C4 levels. Results of the vasculitis workup were negative, excluding vasculitis as a cause for her presentation.

**Figure 3:**
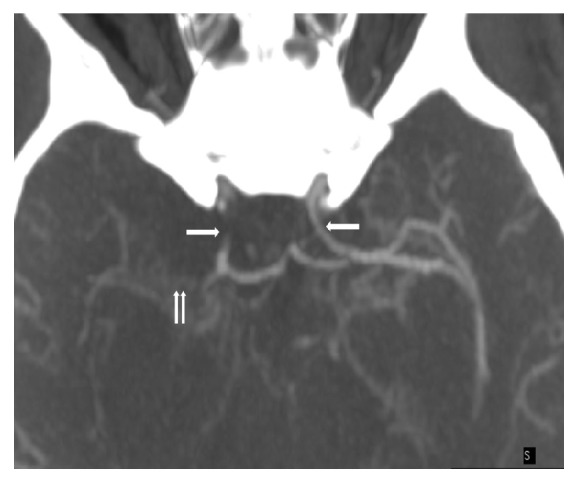
Maximum intensity projection (MIP) axial image of the CT angiogram demonstrates severe stenosis of the internal carotid arteries (single arrows) and occlusion of the Rt, MCA (double arrows).

**Figure 4:**
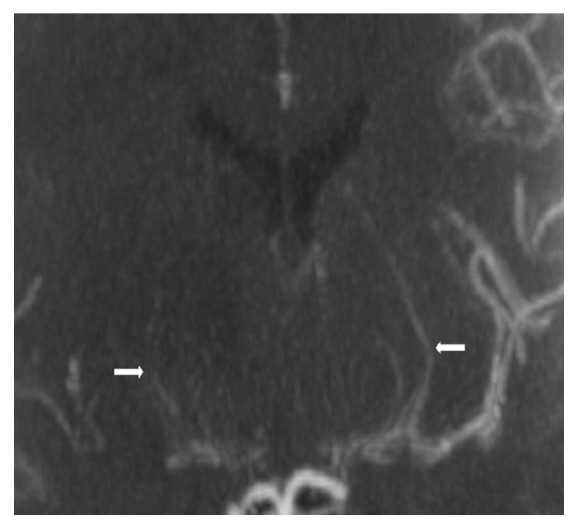
Coronal MIP demonstrates prominent lenticulostriate collaterals in the basal ganglia (arrows).

Brain CT angiography was remarkable for acute bilateral ACA and large right MCA territory infarcts. The intracranial segments of the ICA were diffusely decreased in caliber; there was a complete occlusion of the right MCA from its origin and at least moderate stenosis of the M1 and M2 segments of the left MCA. ACAs were patent but diffusely decreased in caliber, and prominent lenticulostriate vessels were noted in the basal ganglia bilaterally [Figures 3 and 4]. Repeat brain CT the next day showed worsening edema with mass effect on the lateral ventricle and midline shift; thus neurosurgery was consulted and the patient had a left hemicraniectomy. However, mental status remained poor; the patient had a tracheostomy and percutaneous endoscopic gastrostomy and was transferred to a long-term acute care facility.

## 3. Discussion

Acute cerebrovascular events typically present as a constellation of unilateral neurological deficits coinciding to the appropriate unilateral ischemic event. Simultaneous bilateral cerebrovascular infarction is relatively rare [1]. Bilateral ACA territory infarction can be due to vasospasm that occurs as a complication of subarachnoid hemorrhage [2]. Rarer causes of bilateral cerebrovascular infarction can include rupture of an aneurysm of the ACA, thrombus of the precommunicating part of the ACA with an agenesis of the contralateral part [3], or the result of an anomalous unilateral cerebral artery that can mimic a space-occupying lesion [1]. Although transient ischemic attacks (TIAs) and cerebrovascular accidents (CVAs) can typically present with bilateral symptoms as in moyamoya disease, they are not typical. Moyamoya disease in adults commonly presents as either hemorrhagic or ischemic events with ischemic events as the most common presentation in children and adults [4]. Moyamoya disease tends to have a bimodal age distribution affecting children at around 10 years of age and adults aged 30-40 years [5]. In addition to TIA and CVA's, patients with moyamoya disease can present with seizures, headaches, and cognitive impairment. Patients also have a tendency to develop repeated TIAs when they are hyperventilating possibly due to vasodilation of normal vessels and subsequent hypoperfusion in a vulnerable area via steal phenomenon [5]. It is imperative to rule out similar cerebrovascular lesions such as meningitis, brain tumors, TBIs, autoimmune diseases, etc. Diagnosis of moyamoya is a challenge itself due to the rarity of the disease and inclusion criteria as outlined by the research committee on spontaneous occlusion of the circle of Willis (moyamoya disease). The challenge is evident in the acute setting with an initial presentation of symptoms not typical for stroke, as the distribution of neurological deficit was bilateral. An angiogram is recommended at this stage to evaluate intracranial stenosis for possible neurointervention, though it is still controversial. In the SAMMPRIS trial, patients with recent TIA/CVA with intracranial arterial stenosis were questioned if they would benefit with percutaneous transluminal angioplasty and stenting (PTAS) in addition to aggressive medical therapy. Those with PTAS were associated with an increased risk of recurrent stroke when compared to medical therapy alone [6]. Surgical bypass or intracranial angioplasty may offer additional treatment options for moyamoya in the future, though its current efficacy in clinical practice is limited [7].

## 4. Conclusion

Moyamoya disease is a rare clinical syndrome that requires a high index of suspicion to detect. This case presentation outlines an atypical bilateral presentation for an evolving ischemic stroke, with moyamoya disease as the most likely diagnosis. Our findings suggest that, in addition to vasculitis, toxic, and infectious etiologies, moyamoya ischemic pathology should be considered as the cause for a patient to present with altered mental status, sudden onset bilateral upper motor neurological exam findings, and aphasia. We propose that patients with similar presentations be evaluated with an early angiography for evaluation for possible neurointervention, though evidence is currently thin.
